# Renal Function and Ultrasound Imaging in Elderly Subjects

**DOI:** 10.1155/2014/830649

**Published:** 2014-12-04

**Authors:** Luca Zanoli, Giulia Romano, Marcello Romano, Stefania Rastelli, Francesco Rapisarda, Antonio Granata, Pasquale Fatuzzo, Mariano Malaguarnera, Pietro Castellino

**Affiliations:** ^1^Department of Internal Medicine, University of Catania, Via Santa Sofia 78, 95100 Catania, Italy; ^2^Department of Geriatrics, Garibaldi Hospital, 95100 Catania, Italy; ^3^Nephrology and Dialysis Unit, “San Giovanni di Dio” Hospital, 92100 Agrigento, Italy; ^4^Department of Scienze della Senescenza, University of Catania, 95100 Catania, Italy

## Abstract

We evaluated in elderly subjects (a) the ability of GFR formulas to discriminate chronic kidney disease (CKD), (b) the correlation between renal morphology and function, and (c) the usefulness of combined r-US and GFR formulas to detect CKD. A total of 72 patients were enrolled (mean age 80 ± 7 years, male sex 44%, serum creatinine 0.98 ± 0.42 mg/dL, and CKD 57%). Cockcroft-Gault showed the highest sensitivity (78%) and specificity (94%) for CKD and was correlated with kidney volume (*R* = 0.68, *P* < 0.001). All formulas failed to provide a reliable estimate of GFR. In multivariate analysis, Cockcroft-Gault < 52 mL/min and kidney sinus section area < 28 cm^2^ showed the highest accuracy for the identification of CKD subjects (AUC 0.90, *P* < 0.001). MDRD and CKD-EPI differed significantly for GFR ≥90 mL/min.* Conclusions*. Cockcroft-Gault < 52 mL/min was able to discriminate subjects with CKD but all formulas failed to provide a reliable estimate of GFR. The combined use of r-US and Cockcroft-Gault formula improved the ability to discriminate CKD in elderly subjects.

## 1. Introduction and Aims

Only about one-third of the elderly population has a normal glomerular filtration rate (GFR). In these subjects, a reliable estimate of kidney function is of fundamental importance since reduced GFR significantly affects prognosis, drug prescriptions, and dosing [1]. Accurate assessment of renal function requires the measurement of renal clearance of a filtration marker, such as inulin, ^125^iothalamate, ^51^Cr-EDTA, ^99^mTc-DTPA, and iohexol. These measurements, however, are not suitable for routine clinical practice because they are expensive, are time-consuming, and require specialized equipment and skills [2]. In clinical practice, 24-hour creatinine clearance (24 h-CrCl) represents a robust measure of renal function. However, accurate urinary collection in elderly patients may be difficult for the forgetfulness, physical impairment, low compliance, and urinary incontinence. For these reasons, patient's catheterization or caregiver support may be necessary to obtain a correct evaluation of 24-hour creatinine clearance in elderly.

Several formulas based on serum creatinine (SCr) measurements have been developed and tested. The most used formulas in adults are the Cockcroft-Gault (CG, [3]), modification of diet in renal disease (MDRD, [4]), and CKD-EPI [5]. However, the important limit of all of these formulas is that they were derived from data obtained only in adult patients; their performance in other clinical settings, such as in elderly patients, has not been analyzed comprehensively. Previous studies performed in elderly patients reported a poor agreement between predictive equation (CG and MDRD) and more accurate methods were used to evaluate the renal function (24 h-CrCl [6, 7], ^99^mTc-DTPA-GFR [8, 9], or ^51^Cr-EDTA [9, 10]); however, these data are not unanimous [11]. Moreover, in contrast to adult patients [12], MDRD formula seems to overestimate renal function more than CG formula in elderly subjects [13]; the difference between the two formulas increases with age [14]. No data are available about accuracy of CKD-EPI formula in the elderly.

Ultrasound imaging is an easy and widely available technique to evaluate patients in clinical practice. Ultrasound kidney volume and size are reliable predictors of renal function in patients with chronic renal disease [15].

Few data are available on the relationship between kidney morphology and function in elderly subjects. Renal size decreases with increasing age among subjects >60 years [16]. Longitudinal kidney diameter is a determinant of renal prognosis [17]. It is not known whether ultrasound imaging increases the ability of a formula for the estimation of renal function to identify CKD patients. Thus, the present study was designed to evaluate in elderly patients (a) the ability of the most used and validated formulas for the estimation of renal function to identified CKD subjects, (b) the correlation between renal ultrasound parameters and renal function, and (c) the usefulness of renal ultrasound parameters to improve the ability of formula for the estimation of renal function to detect patients with CKD.

## 2. Methods

*Cross-Sectional Study*. Hospitalized elderly subjects with age 65–100 years, catheterized for at least 24 h, were included in this analysis.

Exclusion criteria were absence of bilateral kidneys visualization at ultrasound examination, acute renal failure, wasting disease, kidney cysts > 2.5 cm, malignancy, and absence of informed consent.

CG, MDRD, and CKD-EPI formulas were tested versus 24 h urinary creatinine clearance (24 h-CrCl). CKD is defined as 24 h-CrCl <60 mL/min.

The following kidneys ultrasound parameters were evaluated: mean resistance index (mean-RI), kidney longitudinal diameter (l-diameter), kidney transverse diameter (t-diameter), total kidney volume (t-volume), kidney parenchymal volume (p-volume), parenchymal thickness (mPT), total kidney section area (ASMT), kidney sinus section area (ASMS), and kidney parenchymal section area (ASMP). t-volume was measured by use of the ellipsoid formula (volume = length ∗ width ∗ thickness ∗ *π*/6/2).

Ultrasound analysis was performed by one expert operator (R.M.) with a MyLab 25 device (Esaote, Genova) paired with a 3.5–5 MHz convex probe in transversal and longitudinal sections. Each measurement was performed twice; the mean of the two measurements was recorded.

The study has been conducted in conformity with the ethical guidelines of our institution; an informed consent was obtained from all participants.

### 2.1. Statistical Analyses

All calculations were made using a standard statistical package (SPSS for Windows Version 16.0; Chicago, IL, USA). Continuous variables were reported using means and standard deviations (SD). Categorical variables were described as counts and percentages. The clinical characteristics of patients were compared using analysis of variance for continuous variables and chi-square test for categorical variables. Spearman's correlation coefficient was calculated (*P* < 0.05 was taken as a significant value) to study the relationship between CG, MDRD, and CKD-EPI.

The variability of the measurement of renal function with different techniques was studied using Bland-Altman analysis [18]. The precision was expressed as the width between the 95% limits of agreement (±1.96 SD). Accuracy (Ac) was measured as the percentage of estimated GFR not deviating more than 15%, 30%, and 50% from 24 h-CrCl.

Independent predictors of CKD were identified by univariate and multiple logistic regression analysis considering a set of well-known risk factors for CKD (age, gender, diabetes, hypertension, body mass index, serum creatinine, estimated renal function, and ultrasound parameters). To obtain a parsimonious model, we selected variables that differed (*P* < 0.10) between patients with and without CKD (Table 1). These variables were then jointly included into a multiple logistic regression model and significant, independent predictors of renal dysfunction were identified by a backward elimination strategy. One model was created for each estimating renal function formula. Predictive variables were entered into the model after recodification in binary terms according to the corresponding best cut-off (that is, the value that maximizes the difference between sensitivity and 1 − specificity) in the ROC curves analysis.

The overall discriminatory power for CKD of variables included into the final logistic regression model was investigated by calculating the area under the ROC curve (AUC, [19]) and the corresponding calibration performance was investigated by the Hosmer-Lemeshow goodness-of-fit test [20]. Data are expressed as odds ratio (OR), accuracy, sensitivity, specificity, positive predictive value (PV+), and negative predictive value (PV−).

## 3. Results

The study population included 72 patients (32 males and 40 females). Their demographics and characteristics are summarized in Table 1.

The best cut-off of CG, MDRD, and CKD-EPI for 24 h-CrCl <60 mL/min was 52 mL/min, 87 mL/min, and 65 mL/min, respectively.

**Figure 1:**
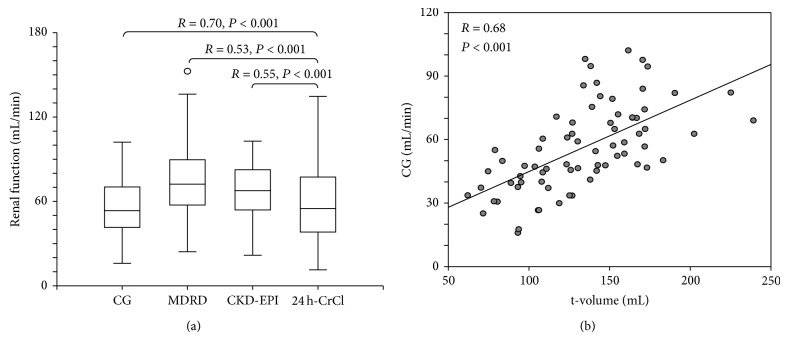
(a) Correlations between formulas for the estimation of renal function and 24 h-CrCl. (b) Correlation between the sum of left and right kidney volumes (t-volume) and renal function estimated by Cockcroft-Gault formula.

**Figure 2:**
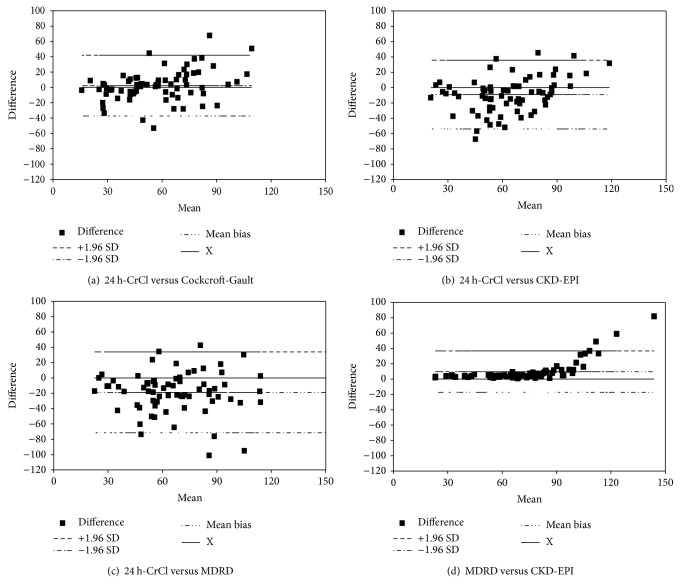
Bland-Altman plot.

The strongest correlation was found between 24 h-CrCl and CG (*R* = 0.70, *P* < 0.001, Figure 1(a)). Bland-Altman analysis of CG revealed a wide SD (20.2 mL/min) around the mean absolute bias (2.3 mL/min, Table 2). Bland-Altman plot is reported in Figure 2. Results of MDRD formula were comparable with CKD-EPI for GFR <90 mL/min and overestimate the renal function for GFR ≥90 mL/min (corresponding to a serum creatinine 0.36–0.64 mg/dL) (Figure 2(d)).

CG showed a high sensibility (78.0%) in selecting patients with CKD and a very high specificity (93.5%) in detecting patients without CKD. Thirty-one patients had a 24 h-CrCl ≥60 mL/min; yet using the CG equation, thirty-eight patients were determined to have GFR ≥60/mL/min with a PV−  of 76.3%. Predictive performance of GFR formulas for CKD is reported in Table 3.

GFR was correlated to kidneys ultrasound parameters (Table 4). The strongest correlation was reported between CG and t-volume (*R* = 0.68, *P* < 0.001; Figure 1(b)).

CG < 52.8 mL/min had the highest accuracy (among the three equations compared) in correctly identifying cases with 24 h-CrCl <60 mL/min. Cox and Snell *R* square was 0.44. The correspondent area under the ROC curve (AUC) was 0.86 (95% CI 0.77–0.95, *P* < 0.001) (Table 5).

In multivariate analysis for 24 h-CrCl <60/mL/min, according to the best cut-off, the following models were selected:

 Model 1: CG < 52 mL/min and ASMS < 28 cm^2^;
 Model 2: MDRD < 87 mL/min, age, and t-volume < 124 mL;
 Model 3: CKD-EPI < 65 mL/min, age, ASMS < 28 cm^2^, and t-volume < 124 mL.

The ROC curves illustrated that Model 1 had the highest accuracy (among the three models) in correctly identifying cases with 24 h-CrCl <60 mL/min. Cox and Snell *R* square was 0.48; the Hosmer-Lemeshow test of the regression analysis was not significant (*P* = 0.80) indicating that the model is well calibrated. The correspondent AUC was 0.91 (95% CI 0.84–0.98, *P* < 0.001). The AUC of each model was reported in Table 5.

## 4. Discussion

Renal function has a significant impact on survival of elderly patients. K-DOQI guideline recommends to estimate the GFR in all subjects >60 years. The reference technique for the evaluation of renal function is the inulin clearance. However, this test is far too complex for regular clinical use. 24 h-CrCl is a robust alternative for the evaluation of renal function in clinical practice but it is difficult to be employed routinely in elderly patients mostly for their low compliance and the need of extra care. Over the years, several equations have been developed for the evaluation of renal function from readily available variables. However, most of these equations, and in particular the recently developed CKD-EPI formula, have not been tested in elderly patients. Therefore, the validation of simple predictive models for CKD, designed to identify patients with 24 h-CrCl <60 mL/min and based on readily available laboratory and morphologic data, may be clinically advantageous in elderly patients.

In the present study, we evaluated the most frequently applied prediction equations for the evaluation of renal function, the CG, MDRD, and CKD-EPI formula. Two of them (MDRD and CKD-EPI) are independent from the determination of body weight which, albeit simple to obtain, may be not readily available in old and frail patients. To assess the suitability of prediction equations, the variability of renal function was evaluated using the Bland-Altman analysis. This test is currently considered superior to the more widely used regression analysis [21]. Our data revealed a considerable lack of precision of all the equations (Table 2). According to the criteria of Ac and precision advocated by the K-DOQI (NKF, 2002), none of the equations had acceptable level of Ac (at least 70%) of estimated renal function within a 30% deviation from 24 h-CrCl. CG is the most accurate formula (Ac 69% within a 30% deviation from 24 h-CrCl). Highest correlation with 24 h-CrCl was obtained by CG (*R* = 0.70, *P* < 0.001). These data are in agreement with the data by Gómez-Pavón et al. [22] who reported a better correlation between CG and 24 h-CrCl than between other formulas and 24 h-CrCl.

The CG equation showed a good sensibility in selecting elderly patients with CKD (Se 78%, Table 1) and had the highest Ac, among the three equations compared, in correctly identifying elderly patients with CKD. From the clinical standpoint, the ability of an equation to identify elderly patients without CKD is also critical. The CG equation was very sensitive in detecting patients without renal dysfunction with a Sp of 94%. The present data are in good agreement with previous reports suggesting a better performance of CG formula in nonobese subjects with normal or marginally impaired renal function.

Our data revealed also a great variability of the estimated renal function between MDRD and CKD-EPI formula for a mean GFR ≥90 mL/min (corresponding to a serum creatinine 0.36–0.64 mg/dL) (Figure 2(d)). This finding suggests that MDRD should be used carefully in elderly subjects with low serum creatinine levels for the risk of a great overestimation of renal function, in particular when a drug prescription and dosing are required.

Ultrasound imaging is a widely available diagnostic procedure that provides much valuable real-time information on the clinical status of elderly patients. A recent study reported a good correlation between total kidney volume evaluated by ultrasound imaging and renal function in patients with chronic kidney disease [15]. In elderly patients both renal function and kidney volume are often reduced but the correlation between renal function and renal size has not been evaluated. Our data show that renal function was correlated to several kidney morphologic parameters (Table 4) and that the strongest correlation was present between CG and t-volume (*R* = 0.68, *P* < 0.001; Figure 1(b), Table 4).

As intrarenal vascular compliance diminishes and subclinical chronic ischemia ensues, the kidneys become smaller. The ratio of ultrasound determined renal volume to intraparenchymal resistive index as evaluated by Doppler at the level of the intrarenal arteries has been proposed as a way to measure nephroangiosclerosis and has been shown to be useful in identifying patients with preclinical hypertensive renal damage, characterized by reduced kidney volume and increased renovascular stiffness [23]. In the present paper we reported that the renal volume/resistive index ratio is correlated to renal function in elderly subjects (Table 4).

We hypothesized that ultrasound kidney parameters may improve the ability of prediction formulas to discriminate elderly patients with CKD. Multivariate logistic regression and ROC curve analysis were performed and three separate prediction models were generated. Among them, Model 1 (CG < 52 mL/min and ASMS < 28 cm^2^) had the highest discriminatory power in identifying CKD patients with an excellent AUC (Table 5). Thus, the present study provides evidences that the combined use of two independent methods (estimating formulas and kidney ultrasound data) may be advantageous in the identification of elderly patients with CKD. Results were qualitatively similar if the renal volume was replaced by the renal volume/resistive index ratio in Models 1–3 but with smaller improvement of AUC.

Our data support the use of formulas for the estimation of renal function and kidney ultrasound parameters for the discrimination of subjects with CKD. When the patients' body weight is unavailable, as it may occur at the bedside of frail elderly patients, CKD-EPI formula can be employed. At this regard, ultrasound-derived kidney parameters (Model 3) will improve the ability of CKD-EPI formula to discriminate between elderly subjects with and without CKD providing a good AUC. Alternatively, if data of body weight are available, the use Cockcroft-Gault formula and ASMS (Model 1) will provide an excellent AUC.

### 4.1. Methodological Issues

A possible limitation of the present study is the absence of gold standard techniques for GFR assessment, such as inulin clearance, in the present study. However, even if the use of 24 h creatinine clearance as reference technique to estimate renal function may have theoretically introduced a bias, the major source of error with the use of this technique is the urine collection which may not reflect the real 24 h urine output [12]. In order to minimize this relevant source of error, the present study was performed in elderly patients in whom a bladder catheter had been placed for another medical reason. This allowed a more reliable estimate of 24 h urine output and minimized the bias. Moreover, due to creatinine excretion via renal tubular secretion, the measurement of 24 h creatinine clearance could be an inaccurate measure of GFR in presence of renal diseases and certain drugs.

## 5. Conclusions

We tested the Ac and the precision of the most common formulas for the evaluation of renal function in elderly patients and evaluated the role of renal ultrasound imaging to improve the ability of prediction formulas to discriminate elderly patients with CKD.

None of the equations reached an acceptable Ac (70%). Cockcroft-Gault provided the best performance (Ac 69%) and had the highest discriminatory power in detecting elderly patients with CKD. The use of renal ultrasound parameters improves the ability of prediction formulas to discriminate between subjects with and without CKD.

## Figures and Tables

**Table 1:** Demographic and clinical characteristics.

	Whole population (*n* = 72 patients)	CKD (*n* = 41 patients)	Non-CKD (*n* = 31 patients)	*P*
Age, years	80 ± 7	82 ± 6	77 ± 7	0.01
Male sex, %	44	42	48	0.56
Weight, Kg	64.9 ± 14.5	61.5 ± 13.2	69.4 ± 15.1	0.09
Height, m	1.58 ± 0.11	1.57 ± 0.11	1.59 ± 0.10	0.42
BMI, Kg/m^2^	25.9 ± 5.6	24.9 ± 5.8	27.3 ± 5.2	0.26
BSA, m^2^	1.70 ± 0.22	1.65 ± 0.21	1.77 ± 0.22	0.08
Diabetes, %	29	22	39	0.12
Hypertension, %	81	76	87	0.22
Serum creatinine, mg/dL	0.98 ± 0.42	1.09 ± 0.47	0.84 ± 0.27	0.01
24 h-CrCl, mL/min	59 ± 27	40 ± 14	84 ± 18	<0.001
Cockcroft-Gault, mL/min	56 ± 20	46 ± 16	70 ± 17	<0.001
MDRD, mL/min	77 ± 29	68 ± 27	90 ± 28	0.001
CKD-EPI, mL/min	68 ± 20	60 ± 19	78 ± 29	0.001
l-diameter, mm	104 ± 16	100 ± 16	110 ± 14	0.003
t-diameter, mm	47 ± 5	46 ± 5	48 ± 5	0.04
t-volume, mL	133 ± 37	117 ± 33	154 ± 31	<0.001
p-volume, mL	95 ± 27	84 ± 26	109 ± 21	<0.001
mPT, mm	6.1 ± 0.8	6.6 ± 0.6	5.8 ± 0.8	<0.001
ASMS, cm^2^	16 ± 4	14 ± 4	18 ± 4	<0.001
ASMT, cm^2^	42 ± 9	38 ± 8	47 ± 7	<0.001
ASMP, cm^2^	26 ± 6	24 ± 6	29 ± 4	<0.001
RI, %	72.8 ± 5.9	73.0 ± 6.4	72.6 ± 5.1	0.68
t-volume/BSA, mL∗m^2^/Kg	79 ± 20	71 ± 18	88 ± 17	<0.001
t-volume/RI, mL	184 ± 49	162 ± 45	213 ± 39	<0.001
t-volume/BSA/RI, mL∗m^2^/Kg	108 ± 28	98 ± 27	122 ± 25	<0.001

BSA, body surface area; ASMS, mean kidney sinus section area; ASMT, mean total kidney section area; ASMP, mean kidney parenchymal section area; l-diameter, mean kidney longitudinal diameter; t-diameter, mean kidney transverse diameter; t-volume, mean total kidney volume; p-volume, mean kidney parenchymal volume; mPT, mean parenchymal thickness; RI, mean resistance index.

**Table 2:** Overall performance of difference and accuracy between 24 h-CrCl and estimated GFR.

Equations	Mean of difference ± SD (mL/min)	*R* ^2^	Accuracy within		
			15%	30%	50%
Cockcroft-Gault	2.3 ± 20.2	0.49	46	69	88
MDRD	−18.8 ± 26.9	0.28	25	44	72
CKD-EPI	−9.1 ± 22.9	0.30	33	56	75

**Table 3:** Diagnostic tests of three estimating GFR formulas for CKD.

Patients with	Se (%)	Sp (%)	PV+ (%)	PV− (%)
CG <52 mL/min	78	94	94	76
MDRD <87 mL/min	88	55	72	77
CKD-EPI <65 mL/min	54	87	85	59

**Table 4:** Spearman's correlation between kidneys ultrasound data and renal function.

	24 h-CrCl	CG	MDRD	CKD-EPI
l-diameter	0.44^b^	0.50^b^	0.30^a^	0.32^a^
t-diameter	0.28^a^	0.44^b^	0.18	0.19
t-volume	0.53^b^	0.68^b^	0.33^a^	0.36^a^
p-volume	0.54^b^	0.66^b^	0.34^a^	0.37^a^
mPT	0.46^b^	0.49^b^	0.27^a^	0.29^a^
ASMT	0.53^b^	0.60^b^	0.29^a^	0.31^a^
ASMS	0.30^a^	0.37^a^	0.07	0.09
ASMP	0.56^b^	0.61^b^	0.34^a^	0.36^a^
RI	−0.03	−0.00	−0.14	−0.16
t-volume/BSA	0.47^b^	0.56^b^	0.50^b^	0.52^b^
t-volume/RI	0.51^b^	0.67^b^	0.37^a^	0.41^b^
t-volume/BSA/RI	0.44^b^	0.55^b^	0.53^b^	0.55^b^

^a^ *P* < 0.05; ^b^ *P* < 0.001. ASMS, mean kidney sinus section area; ASMT, mean total kidney section area; ASMP, mean kidney parenchymal section area; l-diameter, mean kidney longitudinal diameter; t-diameter, mean kidney transverse diameter; t-volume, mean total kidney volume; p-volume, mean kidney parenchymal volume; mPT, mean parenchymal thickness; RI, mean resistance index.

**Table 5:** ROC curve analysis for CKD (24 h creatinine clearance <60 mL/min).

Test result variable(s)	Area (95% CI)	*P*
CG <52 mL/min	0.86 (0.77–0.95)	<0.001
MDRD <87 mL/min	0.71 (0.59–0.84)	0.002
CKD-EPI <65 mL/min	0.70 (0.58–0.83)	0.003
Model 1	0.91 (0.84–0.98)	<0.001
Model 2	0.87 (0.79–0.95)	<0.001
Model 3	0.89 (0.81–0.97)	<0.001
